# Walnut genome editing: an optimized CRISPR/Cas9 platform with superior genotypes and endogenous promoters

**DOI:** 10.1093/hr/uhaf187

**Published:** 2025-06-24

**Authors:** Li Song, Pu Zhang, Ruixue Gao, Baoxin Li, Zhimin Zheng, Xiaobo Song, Dong Pei

**Affiliations:** State Key Laboratory of Tree Genetics and Breeding, College of Forestry, Northeast Forestry University, Harbin 150040, China; State Key Laboratory of Tree Genetics and Breeding, Key Laboratory of Tree Breeding and Cultivation of the State Forestry and Grassland Administration, Research Institute of Forestry, Chinese Academy of Forestry, Beijing 100091, China; State Key Laboratory of Tree Genetics and Breeding, Key Laboratory of Tree Breeding and Cultivation of the State Forestry and Grassland Administration, Research Institute of Forestry, Chinese Academy of Forestry, Beijing 100091, China; State Key Laboratory of Tree Genetics and Breeding, Key Laboratory of Tree Breeding and Cultivation of the State Forestry and Grassland Administration, Research Institute of Forestry, Chinese Academy of Forestry, Beijing 100091, China; State Key Laboratory of Tree Genetics and Breeding, Key Laboratory of Tree Breeding and Cultivation of the State Forestry and Grassland Administration, Research Institute of Forestry, Chinese Academy of Forestry, Beijing 100091, China; State Key Laboratory of Tree Genetics and Breeding, College of Forestry, Northeast Forestry University, Harbin 150040, China; State Key Laboratory of Tree Genetics and Breeding, Key Laboratory of Tree Breeding and Cultivation of the State Forestry and Grassland Administration, Research Institute of Forestry, Chinese Academy of Forestry, Beijing 100091, China; State Key Laboratory of Tree Genetics and Breeding, Key Laboratory of Tree Breeding and Cultivation of the State Forestry and Grassland Administration, Research Institute of Forestry, Chinese Academy of Forestry, Beijing 100091, China

## Abstract

Walnut (*Juglans regia* L.) is an economically valuable tree species globally, renowned for its nutritious nuts and quality timber. However, walnut breeding is significantly constrained by inherent biological factors, and an efficient and reliable genome-editing system has yet to be established. In this study, we developed an optimized walnut genome-editing platform by systematically selecting superior receptor from 30 walnut cultivars using genotype-dependent direct somatic embryogenesis and regeneration systems. Walnut cultivar HT-14 exhibited the highest embryogenic induction (53.33%) and regeneration efficiency (85.33%), and 35S: RUBY was effectively expressed in somatic embryos of the HT-14 genotype, proving it ideal receptor material for genetic transformation. Additionally, 12 walnut-specific endogenous Pol III promoters (JrU3 and JrU6) were cloned and validated for their ability to significantly enhance CRISPR/Cas9-editing efficiency by targeting the walnut phytoene desaturase gene (*JrPDS*). Compared to commonly used exogenous promoters (AtU6-26 and BpU6-6), these native promoters, the JrU3-chr3 promoter achieving an editing efficiency of 58.82%, significantly increased mutation efficiencies in walnut. Furthermore, endogenous promoters promoted higher frequencies of homozygous and biallelic mutations and greater mutation diversity. Collectively, this study establishes a robust and efficient genome-editing platform for walnut, providing essential technical support for functional genomics research and accelerating the precision breeding walnut varieties. These findings also offer valuable methodologies and insights into genome-editing applications in other perennial woody plants.

## Introduction

With rapid advances in genomics and molecular biology, genome editing has become an essential tool in plant research and crop genetic improvement [1]. By enabling precise regulation and modification of targeted genes, genome editing facilitates trait-specific enhancement [2]. This approach accelerates gene function analysis while driving significant advancements in crop yield, quality, and stress resistance, thereby transforming contemporary breeding strategies [3–5]. Among the various available genome-editing tools, the CRISPR/Cas9 system has emerged as a cornerstone of plant research, owing to its exceptional efficiency, high specificity, and operational simplicity [6]. Derived from bacterial adaptive immune mechanisms, this system uses single-guide RNA (sgRNA) to guide the Cas9 nuclease to precise loci in the target genome, where it induces double-strand breaks. These breaks are subsequently repaired by cellular mechanisms via either nonhomologous end joining or homology-directed repair pathways, enabling gene knockout, insertion, or replacement [7]. This efficient and precise editing mechanism has been extensively applied in crop genetic improvement and functional genomics research, offering transformative potential for plant science [8].

The CRISPR/Cas9 system has been extensively and successfully applied to improving various economically important crops. For instance, editing disease-resistance genes in rice has significantly enhanced pathogen resistance [9]. Similarly, targeting drought-resistance genes in wheat has increased drought tolerance [10], and modifying fruit-ripening-related genes in tomatoes has improved fruit quality [11]. Beyond crop improvement, CRISPR/Cas9 plays a critical role in plant functional genomics. By enabling precise editing of target genes, it facilitates the elucidation of gene functions related to plant growth, development, and stress responses [12, 13]. Collectively, these advancements provide a robust theoretical foundation and critical technological support for future crop genetic enhancement.

However, establishing genome-editing systems for woody plants remains significantly challenging [14]. One primary obstacle is the underdevelopment of *in vitro* culture and regeneration systems. Somatic embryogenesis and regeneration in woody species are highly dependent on genotype, tissue type and culture conditions [15]. These factors frequently lead to tissue browning and low-regeneration efficiency, limiting the availability of suitable receptor material [16, 17]. Another challenge is the inherently low efficiency of genetic transformation. The thick cell walls and strong genotype dependency of woody plants hinder the effectiveness of traditional transformation methods, such as Agrobacterium-mediated transformation and gene gun techniques [15, 18]. These methods often result in unstable expression of exogenous DNA and gene silencing. A further bottleneck is the absence of robust gene-expression regulatory systems. Genome editing in woody plants typically relies on exogenous promoters, such as CaMV 35S and U6, to drive the expression of Cas9 and gRNA. However, these promoters often demonstrate poor compatibility with woody plant genomes and are susceptible to host-mediated transcriptional regulation or DNA methylation, leading to unstable or silenced expression [19]. For example, in *Physcomitrella patens*, the rice OsU3 promoter resulted in only 11.1% full mutations, while the endogenous PpU6 achieved 91.4% [20]. In soybean hairy roots, editing efficiency rose from 3.2%–9.7% with AtU6 to 14.7%–20.2% using GmU6 [21]. Similarly, EgU6–2 in *Eustoma grandiflorum* outperformed AtU6 by over 30% [22]. In maize, some heterologous U6 promoters showed mutation rates as low as 0.018% [23]. These findings underscore the limitations of heterologous U6/U3 promoters in perennials and highlight the importance of developing species-specific endogenous promoter systems to improve editing precision and efficiency. Endogenous promoters, more compatible with host genomes, offer potential advantages for achieving stable and efficient gene expression. Recent studies have begun to identify and apply endogenous promoters from woody plants to improve gene-editing efficiency [24]. In white birch, using the phytoene desaturase (PDS) as the target gene, 16 endogenous Pol III promoters were identified and screened, leading to the establishment of an efficient genetic transformation and gene-editing system [25]. In grapevine, four endogenous VvU3 and VvU6 promoters, along with two ubiquitin promoters, were identified. The use of these endogenous promoters significantly enhanced editing efficiency by improving the expression of sgRNA and Cas9 [26].

Walnut (*Juglans regia* L.), an economically significant tree species worldwide, is highly valued for its nutrient-rich nuts and high-quality timber [27]. China is the world's largest producer of walnut, possessing abundant germplasm resources that encompass a wide range of desirable traits, including early maturation, high yield, and elevated oil and protein content. These diverse genetic resources provide a critical foundation for advancing molecular breeding efforts in walnut [28, 29]. Over the past decade, research has advanced the genetic dissection of important traits, the development of high-performing cultivars and the optimization of clonal propagation systems [30]. However, traditional breeding in walnut remains constrained by a long juvenile phase, high genomic heterozygosity, and limited transformation efficiency, all of which prolong the breeding cycle and reduce the feasibility of integrating multiple favorable traits. These limitations underscore the urgent need for efficient, precise, and genotype-flexible breeding tools to accelerate cultivar development.

CRISPR/Cas9-based genome editing offers a promising solution for targeted walnut improvement, particularly in the context of available high-quality genome assemblies that facilitate the identification of functional genes and sgRNA design [31]. However, existing editing systems in walnut predominantly rely on exogenous promoters borrowed from model species to drive Cas9 and gRNA expression [32]. These elements frequently result in low transcriptional activity, weak tissue specificity, and instability, ultimately compromising editing efficiency. Moreover, editing success is strongly influenced by the receptor genotype, as walnut germplasms vary widely in their embryogenic capacity, regenerative potential, and transformation responsiveness. While some genotypes support efficient regeneration, others exhibit issues such as tissue browning, poor proliferation, and low DNA uptake. Accordingly, overcoming these technical constraints necessitates the development of a walnut-specific genome-editing platform that leverages endogenous regulatory elements and high-performing receptor genotypes, thereby ensuring efficient, stable, and genotype-adapted editing results.

This study aimed to establish an efficient genome-editing system for walnut, with a focus on selecting superior receptor materials and developing and validating walnut-specific endogenous promoters. We systematically assessed *in vitro* induction, regeneration capacity, and transformation efficiency across diverse walnut germplasms, ultimately identifying receptor materials suitable for high-efficiency editing. Notably, this research was the first to develop and validate the genome-editing efficiency of endogenous U3 and U6 promoters in walnut. Together, these efforts led to the establishment of a robust and efficient genome-editing platform, offering essential technical support for walnut genomics, functional gene studies, and precision breeding. Furthermore, it offers novel insights and valuable technical references for genome-editing research in other economically significant tree species, contributing to the modernization and sustainable development of forestry.

## Results

### Somatic embryogenesis in different walnut variations

**Figure 1:**
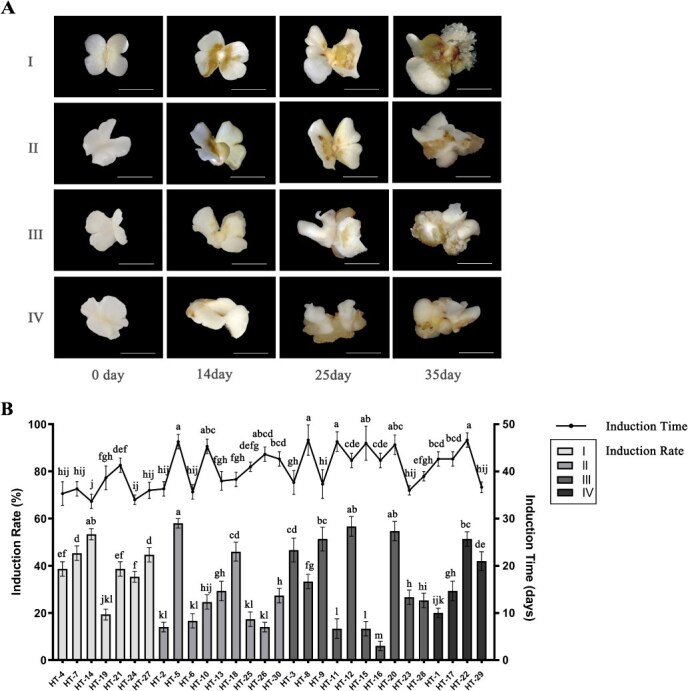
Induction of SEs by zygotic embryos in walnut. A. SE induction process: Type I: SEs formed at the embryo tip and cotyledon surface without callus formation. Type II: Callus formed during induction, with SEs developing on the cotyledon surface. Type III: Callus appeared during induction, and a few SEs formed around the embryo tip. Type IV: Callus-free tissue formed during induction, with a small number of SEs appearing on the cotyledon surface at later stages. B. Comparison of induction efficiencies and induction durations for immature embryos across 30 walnut cultivars. The bar graph represents the induction rate, and the colors at different depths correspond to the four categories in A; the line graph represents the time required for induction to produce SEs. Results show mean ± SD of three repeat events. Error bars indicate SD. *P* < 0.05 (Tukey HSD). Bar = 0.5 cm.

To establish an efficient genetic transformation system, optimal receptor materials were first identified. A total of 30 walnut varieties exhibiting distinct characteristics were selected from six regions in China, including: Xinjiang, Tibet, Henan, Hebei, Beijing, and Liaoning (Supplementary Table S1). At the optimal developmental stage (approximately 50 days after peak female flowering), 50 immature embryos from each cultivar were collected and cultured under identical induction conditions for comparative analysis. The 30 walnut genotypes exhibited distinct somatic embryo (SE) responses, which were categorized into four types based on induction timing and embryogenic pathways (Fig. 1A and B). Type I, 7 lines such as HT-14 and HT-24 were direct somatic embryogenesis, with globular embryos emerging around 25 days postinduction (dpi) and fully structured cotyledonary embryos forming by approximately 35 dpi, reflecting strong embryogenic potential. Types II and III followed an indirect SE pathway, with callus formation around 25 dpi and cotyledonary SEs forming at approximately 45 dpi. Callus growth persisted, accompanied by browning and necrosis, indicating stress during induction. Type III exhibited significantly prolonged induction times, with some varieties (eg, HT-8, HT-11) requiring more than 45 days to form complete SEs, showing clear delays (Supplementary Fig. S1). Type IV had the lowest SE induction, with limited embryo formation and extended induction time.

**Figure 2:**
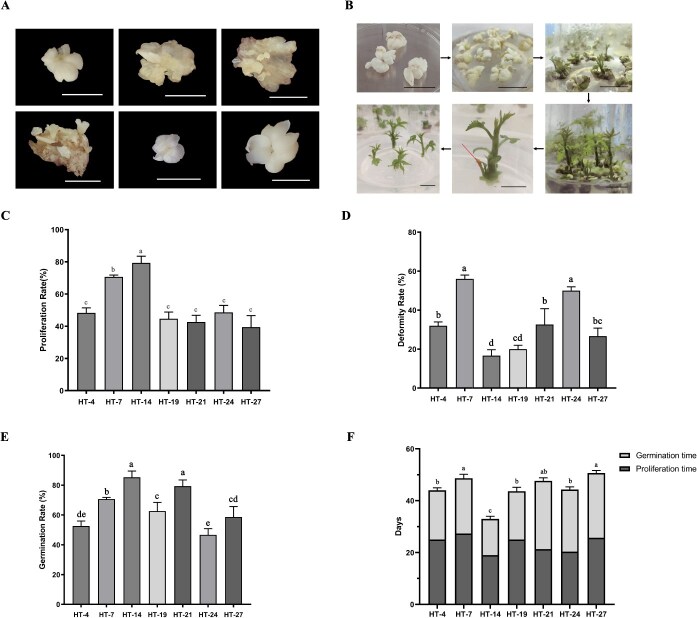
Evaluation of the SEs capacity in walnut. A. The proliferative process of SEs in walnut. B. The process of transformation and seedling formation of SEs in walnut. C. Comparison of proliferation rates of SEs. D. Comparison of the deformity rate of newborn embryos. E. Comparison of germination efficiency during SE transfer. F.30 Statistics of regeneration time of individual SE material. Results show mean ± SD of three repeat events, 50 SEs were selected for each event. Error bars indicate SD. *P* < 0.05 (Tukey HSD). Bar = 0.5 cm.

Comparison of SE induction rates among the four embryo types revealed significant differences (Fig. 1C). Varieties in Type I generally exhibited higher induction rates (average ~ 40%) compared to the other three types (*P* < 0.05). Specifically, HT-14 showed the highest induction rate (53.33%), followed by HT-7 (45.33%) and HT-27 (44.67%). In contrast, Type II and Type III embryos exhibited generally lower induction rates but significant variability within groups. For instance, within Type II, variation HT-5 displayed a higher induction rate notably, while within Type III, HT-12, and HT-20 showed significantly elevated induction rates compared to other varieties. Conversely, HT-19 (Type I) exhibited a substantially lower induction rate compared to most varieties in other categories. In this study, we observed significant differences in somatic embryogenesis among the 30 varieties, which may be attributed to genotype-specific varieties. Genotype affects both the capacity and the pathway of somatic embryogenesis. Based on the direct somatic embryogenesis and strong embryogenic response in Type I, we further compared somatic embryogenesis regeneration capacity among different genotypes within Type I.

### SE regeneration ability in different walnut varieties

Efficient regeneration of complete plantlets from SEs is critical for the successful establishment of genetic transformation systems [33–35]. In this study, an efficient plant regeneration system from somatic embryos (SEs) was successfully established. Seven walnut genotypes (HT-4, HT-7, HT-14, HT-19, HT-21, HT-24, and HT-27), which had previously been identified as having high germination potential, were selected for evaluation. Their regeneration capacities were systematically assessed and compared, including parameters such as embryo proliferation rate, deformity rate, germination rate, and the total duration required for the regeneration cycle (Fig. 2). Figure 2A illustrates the proliferation and regeneration process of walnut SEs. The newly formed embryos emerge from the cotyledon surface of the SEs and can be separated from the cotyledon surface and develop into mature SEs. After dehydration treatment, the mature SEs undergo germination under light conditions, eventually growing into walnut seedlings (Fig. 2B), completing the entire walnut tissue culture regeneration process.

Among the seven walnut varieties analyzed, significant genotype differences were observed in SE proliferation rate (Fig. 2C), deformity rate (Fig. 2D), germination rate (Fig. 2E), and regeneration time (Fig. 2F). HT-14 exhibited the strongest overall regeneration ability, with the highest SE proliferation rate (79.33%), the lowest deformity rate (16.67%), the highest germination rate (85.33%), and the shortest regeneration cycle (33 days). Although HT-7 showed high proliferation and good germination capacity, its deformity rate was the highest (56%) and regeneration cycle was longer. HT-4 had a deformity rate higher than 50%. HT-21, while having a high germination rate, had weaker proliferation capacity. HT-24 had a high deformity rate and a low germination rate. HT-27 performed the worst, with the lowest proliferation rate and the longest regeneration cycle. HT-19 performed slightly better than HT-27, with a lower deformity rate, but its proliferation ability was weak. Collectively, genotype HT-14 demonstrated superior performance with the highest embryo proliferation and germination rates, the lowest deformity rate, and the shortest regeneration cycle duration. Based on these findings, HT-14 was selected as the optimal receptor material for subsequent genetic transformation studies in walnut.

### 35S: RUBY transgene SEs of HT-14

**Figure 3:**
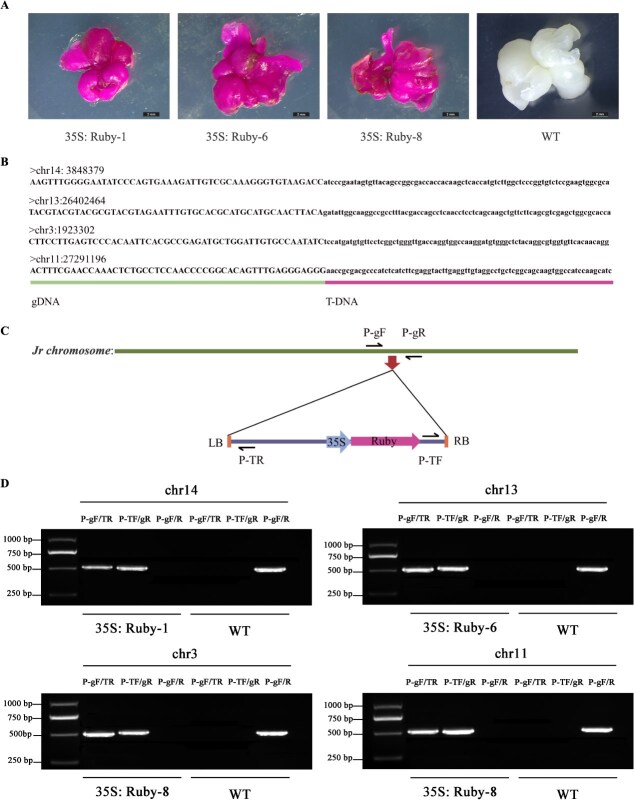
Establishment of a visualized genetic transformation system in walnut. A. Random selection of three Ruby phenotype transgenic SEs and one WT HT-14 SE. B. The T-DNA insertions on the chromosomes of the walnut genome were analyzed by resequencing. The walnut genome DNA sequence is shown in majuscule letters, and the T-DNA sequence is shown in lowercase letters. C. Location of T-DNA insertion and detection primers in walnut genome. D. PCR detects T-DNA insertions into chromosome genomes, with P-gF/R corresponding to the respective line primers, in Supplementary Table S5. Bar = 2 mm.

The stable integration of exogenous genes into the plant genome is critical for consistent genetic expression [36]. To evaluate the suitability of SE HT-14 as transformation recipients, we employed the RUBY gene, which produces a visible red pigment in transformed tissues, serving as a convenient visual marker [37]. At three days postinfection, transient light-red expression appeared in young secondary somatic embryo epidermal cells (Supplementary Fig. S2A), fading within a week. This transient transformation occurred at 13.33% (Supplementary Fig. S2B), with no recurrence in subsequent subcultures. The stable transformation efficiency was 53.33%, showing distinct deep-red somatic embryos. And the RUBY phenotype remained stable in new embryos through multiple subcultures (Supplementary Fig. S2C). To detect exogenous gene insertion, we randomly selected three somatic embryos with RUBY phenotype from E_2_ generation and one white WT of HT-14 (Fig. 3A) for *p35S: RUBY* T-DNA insertion analysis using resequencing.

The whole-genome sequencing data of the three transgenic walnut plants exhibited high quality, meeting the high-throughput sequencing quality control standards (Supplementary Table S2). Using Bowtie2 with default parameters, sequencing reads were aligned against the 35S: RUBY T-DNA sequence, identifying T-DNA insertion sites in the transgenic lines. Lines 35S: Ruby-1 and 35S: Ruby-6 contained single-copy insertions located at chr14: 3848379 and chr13: 26402464, respectively, while 35S: Ruby-8 displayed two candidate insertion sites at chr3: 1923302 and chr11: 27291196 (Fig. 3B). To further validate these insertion sites, T-DNA-genome border-specific primers were designed (Fig. 3C), and PCR verification was conducted (using the primers of P-gF/P-TR and P-TF/P-gR in Supplementary Table S5). Line 35S: Ruby-8 produced two distinct PCR bands, confirming its double-copy insertion, whereas lines 35S: Ruby-1 and 35S: Ruby-6 each yielded a single band, indicative of single-copy insertions, while the WT showed no amplification. However, using Pg-F/R primers, transgenic lines failed to yield amplification products due to oversized inserts (9092 bp), whereas wild-type materials produced ~500 bp bands (Fig. 3D). These results confirmed the successful integration and stable inheritance of the exogenous T-DNA in the walnut genome.

### Establishment of an endogenous JrU3/JrU6 promoter-based CRISPR/Cas9 platform in walnut

The editing efficiency of CRISPR/Cas9 is influenced by multiple factors, including the target site, species-specific characteristics, and sgRNA transcription. Using species-specific endogenous Pol III promoters can significantly increase the abundance of sgRNA expression, thereby improving genome-editing efficiency [38–41]. To optimize the CRISPR/Cas9 system in walnut, we identified four JrU3 and eight JrU6 snRNA genes in the walnut genome by referencing the AtU6 and AtU3 genes of Arabidopsis. Multiple sequence alignment revealed that the walnut U6 snRNA gene shared over 97% homology with Arabidopsis U6 snRNA, while the homology between walnut U3 snRNA and Arabidopsis U3 snRNA was approximately 80%. Although there were some differences in the promoter regions of walnut snRNA genes, all contained USE and TATA-like boxes, which may effectively drive the transcription of sgRNAs in the gene-editing system (Supplementary Fig. S3 and Supplementary Table S3).

To evaluate the effectiveness of CRISPR/Cas9-mediated gene editing in walnut, we targeted the PDS gene, whose disruption results in a white phenotype in plants [42]. A phylogenetic analysis of the walnut genome identified a single-copy *JrPDS* gene (JreChr01G1095) (Supplementary Fig. S4), which was used as a reporter gene to construct an optimized CRISPR/Cas9 vector (*pSCI-JrU3/U6-PDS*), and using BpU6-6 and AtU6-26 as controls [25]. This vector was used to assess the efficiency of walnut U6/U3 promoters in genome editing (Supplementary Figs S5 and S6). Using Agrobacterium-mediated transformation, the CRISPR vector was successfully introduced into walnut SEs. After antibiotic selection, Basta-resistant SEs were obtained, which were subsequently regenerated into plants. Through PCR-based positive identification, walnut lines carrying the CRISPR/Cas9 vector were preliminarily identified as transgenic, indicating the presence of vector sequences in plant cells.

**Figure 4:**
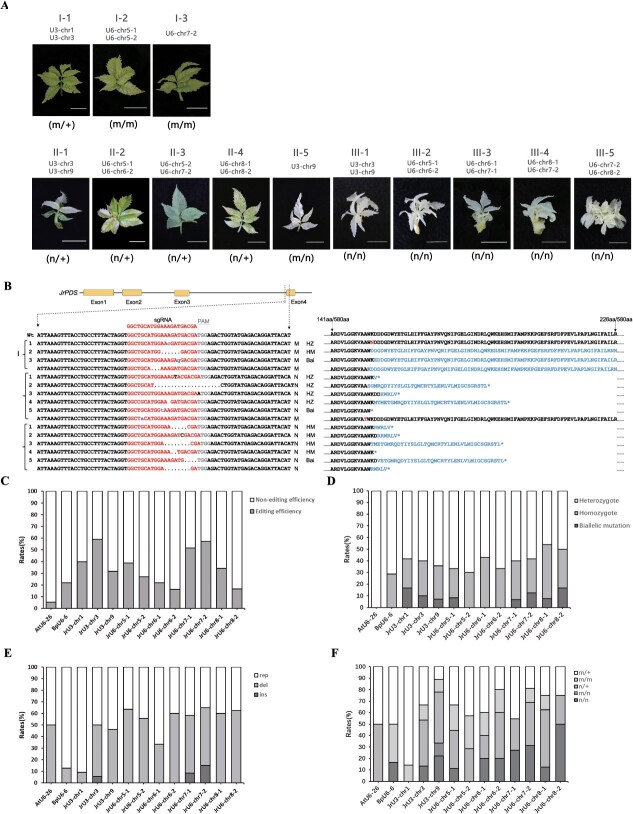
Establishment of a visualized gene-editing system in walnut. A. Phenotypes of transgenic lines induced by control of sgRNA expression by JrU3/U6 promoters in a CRISPR/Cas9 system in walnut. + denotes wild type, s, synonymous mutation, m, missense mutation, and n, nonsense mutation. B. Sequencing results and amino acid sequence changes of *JrPDS* target gene site mutations caused by promoter driven sgRNA expression. C. Discrepancies in genome-diting efficiencies using various promoters to drive sgRNA expression in walnut. Percentages were obtained by dividing the number of edited lines by the total quantity of positive transformants. D. Proportion of heterozygous, homozygous, and biallelic mutations arising from using different endogenous promoters to drive sgRNA expression in walnut. Percentages were calculated by dividing the total number of each type of mutation by the number of edited plants. E. Rates of substitution, insertion, and deletion mutations that arose when using different endogenous Pol III promoters to drive sgRNA expression in walnut. Percentages were calculated by dividing the total number of each mutation type. Rep, replacement; del, deletion; ins, insertion. F. Percentages of plants expressing different protein structure variants when sgRNA expression is driven by various endogenous Pol III promoters in walnut. Percentages were calculated by dividing the total number of each mutation type. Bar = 1 cm.

To further assess the genomic-editing effects of the CRISPR/Cas9 system in transgenic walnut lines and evaluate the impact of different promoters on editing efficiency, we extracted genomic DNA from transgenic positive lines and wild-type lines. PCR amplification was performed on the PDS gene target site and its adjacent regions, followed by enrichment. The obtained PCR products were then purified and subjected to Sanger sequencing to analyze the mutations at the target sites (Fig. 4A and B and Supplementary Figs S7–S9). Phenotypic analysis showed that, except pSCI-JrU3-chr10-PDS, the remaining 13 transformation plasmids successfully produced transgenic plants exhibiting an albino phenotype (Fig. 4A). Sanger sequencing results (Fig. 4C) revealed that the promoters driving sgRNA expression with high editing efficiency were JrU3-chr3 (58.82%), JrU6-chr7-2 (57.14%), and JrU6-chr7-1 (51.72%), with editing efficiencies exceeding 50%. The editing efficiencies of the four other promoters, JrU3-chr1 (40.00%), JrU6-chr5-1 (38.71%), JrU6-chr8-1 (34.21%), and JrU3-chr9 (31.82%), were also above 30%. The editing efficiencies of the remaining promoters were relatively low, with JrU6-chr5-2 (27.03%), JrU6-chr6-1 (21.88%), JrU6-chr8-2 (16.67%), and JrU6-chr6-2 (16.22%) all below 30% (Supplementary Table S4). These results indicate that the endogenous Pol III promoters in the walnut genome exhibit generally higher editing efficiency than the Arabidopsis AtU6-26 promoter (5.41%) and the birch BpU6-6 promoter (21.88%). Based on this study, we established an efficient gene-editing platform for walnut.

In addition, we also found that the CRISPR/Cas9 system driven by endogenous promoters induced mutations with a higher frequency of nonhomozygous mutations in walnut (Fig. 4D). Compared to heterozygous editing, homozygous editing offers higher genetic stability and more pronounced phenotypic changes, making it more suitable for long-term studies and gene function validation. Unlike the transgenic lines driven by the AtU6-26 promoter, where only heterozygous mutations were detected, transgenic lines driven by endogenous promoters exhibited not only homozygous mutations but also double allelic mutations, with a significant increase in the proportion of nonheterozygous mutations. The CRISPR/Cas9 system driven by endogenous promoters induced more diverse types of mutations compared to exogenous promoters (Fig. 4E), primarily including single-base mutations and base deletions, with the number of deleted bases ranging from 1 to 19. Single-base insertions were also observed when using the JrU3-chr3, JrU6-chr7-1, and JrU6-chr7-2 promoters, accounting for 5.55%, 8.33%, and 15% of the total mutation types, respectively. By analyzing mutation types and corresponding changes in protein structure, the mutant lines were classified into three protein variation types: missense-missense (mm, Type I), missense-nonsense (mn, Type II), and nonsense-nonsense (nn, Type III) (Fig. 4F). The results indicate that genotypes containing nonsense mutations (n) were critical for the albino phenotype. When n was heterozygous, a mosaic phenotype in the transgenic plants, whereas homozygous nn leaded to complete albino plants. In contrast, missense mutations (m) contributed less to the albino phenotype, and transgenic plants with either homozygous mm or heterozygous m exhibited a green phenotype. Further analysis showed that transgenic plants with n + and nn genotypes dominated in almost all edited lines. Notably, except for JrU6-chr5-2, transgenic lines driven by endogenous Pol III promoters showed higher proportions of n + and nn genotypes compared to those driven by exogenous promoters such as AtU6-26 or BpU6-6. Only transgenic lines edited with the JrU3-chr9 promoter generated the mn genotype. Additionally, in transgenic lines edited with JrU6-chr8-2, JrU6-chr7-2, JrU6-chr7-1, JrU3-chr9, JrU6-chr6-1, and JrU6-chr6-2 promoters, the proportion of nn genotype plants was significantly higher than in those edited with the BpU6-6 promoter. These results further demonstrated that endogenous Pol III promoters significantly enhanced genome-editing efficiency in walnut, particularly in terms of albino phenotype variation, showing a clear advantage over exogenous promoters such as AtU6-26 and BpU6-6. Overall, the findings strongly demonstrated the effectiveness of using walnut endogenous promoters for gene editing, offering a superior strategy to improve genome-editing efficiency in this species.

## Discussion

### Direct embryogenesis in walnut and its application

Plant tissue culture regeneration enables plant regeneration through the differentiation and development of explants, and is a key technique for functional genomics research and understanding plant development mechanisms. In most plants, regeneration involves dedifferentiation of explants into callus tissue, while some species bypass the callus stage through direct somatic embryogenesis [33, 35, 43]. In this study, we observed that seven walnut varieties from Class I, such as HT-14 can directly form SEs from immature zygotic embryos during induction culture, without undergoing callus formation. This direct embryogenesis differs from the conventional callus-based regeneration pathway and may be strongly associated with the inherent developmental potential of walnut embryonic tissues.

Direct somatic embryogenesis has been reported in various plant species. For example, in peanut, optimized culture conditions allow new embryos to differentiate directly from the zygotic embryo [44, 45]. In cotton, modulation of hormone concentrations in the culture medium can induce direct embryogenesis, bypassing the callus stage [46]. In *Tilia*, SEs are directly induced from immature zygotic embryos and can rapidly differentiate into complete plants under appropriate conditions, offering a rapid propagation strategy [47]. Similarly, in grapevine, distinct somatic embryogenesis responses have been observed across varieties: under identical culture conditions, 'Cabernet Sauvignon' exhibits higher sensitivity and embryogenesis rates, while 'Chardonnay' shows lower efficiency [26]. The capacity for direct somatic embryogenesis is likely closely related to the developmental competence of embryonic tissues. This process not only enhances tissue culture efficiency but also reduces the risk of genetic variation associated with dedifferentiation and cellular reprogramming [35], representing a potential evolutionary strategy for plant adaptation to environmental challenges.

### Somatic regeneration efficiency and genotypes

Furthermore, we explored the effect of genotypes on the regeneration efficiency of SEs. Seven walnut cultivars were tested in this study, with the effect manifesting at multiple stages of regeneration, including induction, proliferation, SE morphology, and plantlet regeneration. This is likely due to differences in the response of plant materials with different genotypes to culture conditions (such as hormone types and concentrations). For example, in rice and maize [43, 48], certain genotypes are more sensitive to 2,4-D and can produce callus tissue with high regeneration potential, other genotypes show lower potential and reduced callus induction ability. Under the same NAA conditions, soybean genotypes such as ‘BS-6’ were found to be better explants than ‘ST’, showing better performance in callus induction and plant regeneration [49]. Furthermore, some genotypes possess endogenous hormone levels and regulatory gene expression patterns that favor direct somatic embryogenesis. In kohlrabi, the differences in organogenesis response between explants of the ‘Vienna Purple’ and ‘Vienna White’ varieties were associated with differences in cytokinin and IAA content [50]. In walnut, the variation in regeneration efficiency among different genotypes is notable, which may be closely related to the genetic background of the embryonic tissues.

The genetic background of the genotype affects the culture ability and regeneration potential of plant materials. For instance, studies in canola [51]and cotton [46] show that specific genotypes exhibit higher embryo regeneration rates under low hormone conditions, while other genotypes require higher hormone concentrations to achieve similar results. More importantly, the genotype also influences the genetic stability and growth characteristics of regenerated plants. Studies in woody plants such as tea trees and *Tilia* [47, 52] reported that genotypes with high regeneration efficiency show better stability during subculture during subculture. Our walnut genotype HT-14 also exhibits high regeneration efficiency, relatively stable somatic embryo morphology, and a low deformity rate of 16.67%. Its high proliferation rate (79.33%) demonstrates strong embryogenic potential, while the low deformity rate ensures the quality and vitality of regenerated embryos. This facilitates the propagation and application of the material, reduces experimental complexity, and promotes its wider use. Additionally, research has shown that genotype is a critical limiting factor in Agrobacterium-mediated transformation [36]. In crops such as maize [53], wheat [54], and barley [55], genotype significantly influences the genetic transformation efficiency between different varieties, and genotype may determine the biochemical mechanisms and processes of differentiation and regeneration through its genetic characteristics.

### Genetic transformation efficiency and receptor material

In plant genetic transformation studies, selecting appropriate receptor material is key to improving transformation efficiency and plant regeneration rates. Common infection receptors include leaves, callus tissue, embryoids, and protoplasts [56]. The leaf disc method, due to its technical maturity and broad applicability, has become the mainstream method for genetic transformation, especially in model crops such as poplar, tobacco [57], and tomato [58]. However, this method is prone to forming chimeras and is greatly influenced by plant genotype and culture conditions. Using immature embryos or nutrient organs-induced callus tissue as infection receptors can reduce the formation of chimeras [59]. Additionally, callus tissue shows sustainable proliferation and high division activity. It is not limited by the initial explant, and is suitable for introducing long gene fragments and supports complex genetic transformations. This approach has been successfully applied in genetic transformation of various woody plants, such as larch [60], tulip tree [61], and eucalyptus [62]. Protoplasts achieve efficient gene transfer by removing the cell wall and are suitable for short-term gene function validation [63]. However, the success rate of regenerating complete plants is relatively low, and their application scope is limited. SEs, on the other hand, are ideal receptor materials for genetic transformation studies due to their uniform genetic background, low chimerism occurrence, and strong regenerative ability [64]. For example, Maleki et al. [65] successfully established an SE transformation system in *Pinus massoniana* by *Agrobacterium* infection after a seven-day dark preculture, without the need for callus induction. Moreover, the direct differentiation of SEs into single-cell-derived regeneration pathways [66] offers significant advantages. Compared to other regeneration methods, this approach can significantly reduce chimerism formation and help maintain homozygosity [67]. However, the induction of SEs is greatly limited by species and genotype, and subsequent regeneration and transformation experiments require complex culture conditions and high technical expertise. These factors restrict the widespread use of SEs as receptor materials in genetic transformation. When selecting receptor materials, it is essential to consider experimental goals, plant characteristics, and technical conditions comprehensively. By rationally selecting receptor materials, transformation efficiency can be maximized, and plant regeneration success rates can be improved, thus advancing plant genetic transformation research.

### Endogenous promoters and genome editing

The advent of the CRISPR/Cas9 system has revolutionized genome editing and gene therapy, compared to traditional genome-editing technologies, CRISPR/Cas9 is simple, precise, and efficient, and has been widely applied in many plant species. In disease resistance breeding, editing the promoter of *CsLOB1* in citrus conferred heritable canker resistance [68], while knockout of *PtoMYB221* in poplar dramatically altered lignin composition [69]. For fruit quality improvement, editing *VvCCD4* in cucumber successfully modulated carotenoid metabolism to create novel flesh-color phenotypes [70], and targeted editing of *MdMYB10* in apple enabled precise anthocyanin accumulation in fruit skin [71]. Developmental engineering breakthroughs reducing the juvenile phase in apple to <12 months by editing *MdTFL1.1* [72] These cases validate CRISPR's pivotal role in overcoming perennial crop breeding constraints.

Despite these successes, efficient and stable editing remains limited by species-specific transformation efficiencies, high levels of chimerism, and variable expression of CRISPR components driven by conventional constitutive promoters such as CaMV 35S or heterologous U6/U3. Emerging evidence highlights the role of endogenous promoters in overcoming major barriers to efficient genome editing. This system uses single-guide RNA (sgRNA) and Cas9 nuclease for genome editing, where the sgRNA guides Cas9 to specific DNA targets through RNA–DNA pairing and causes double-strand DNA breaks. The expression of sgRNA and Cas9 has been shown to impact editing efficiency [73, 74]. Increasing sgRNA expression to enhance gene-editing efficiency is an important strategy for current improvements, and the application of species-specific promoters has been shown to effectively increase sgRNA expression [35]. To date, the eukaryotic U3 and U6 promoters, isolated from rice and Arabidopsis, respectively, have been widely used in monocot and dicot plants [75, 76]. In previous walnut genome-editing studies [77], the AtU6 promoter has commonly been used to drive the expression of sgRNA, respectively.

**Figure 5:**
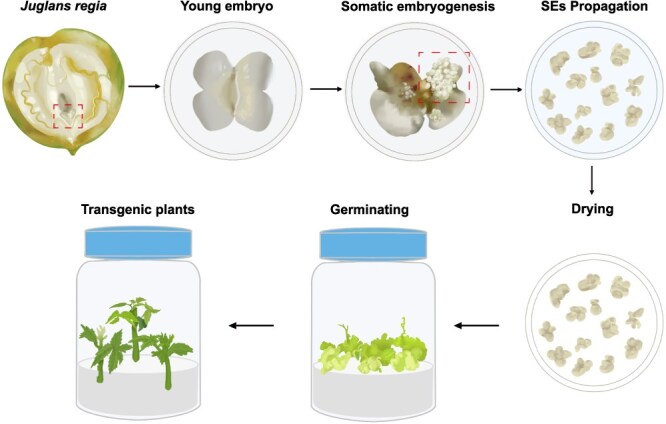
The whole flow scheme of a high-efficiency direct somatic embryogenesis regeneration system in walnut. A protocol for efficient direct somatic embryogenesis system using immature cotyledon explants was developed according to the above results.

Previous research has confirmed that endogenous Pol III promoters, such as the U3 and U6 series, could effectively drive sgRNA expression. This strategy significantly enhances the genome-editing efficiency of the CRISPR/Cas9 system and increases the diversity of mutation types [75]. In this study, we identified 12 endogenous Pol III promoters in walnut and verified their efficiency in walnut genome editing by targeting the *JrPDS* gene. We found that using endogenous promoters (such as JrU3 and JrU6) to drive sgRNA expression significantly improved the editing efficiency of walnut lines. Compared to the AtU6-26 promoter from Arabidopsis and the BpU6-6 promoter from white birch, most improvements exceeded 30%. In various plant species, such as wheat [78], grape [79], and larch [80], using endogenous promoters showed higher editing efficiencies compared to OsU6a. Furthermore, studies in spruce and cotton [40] have further shown that sgRNA expression driven by endogenous promoters is more precise and efficient. The GhU6.3 promoter increased sgRNA expression levels by 6–7 times, and mutation efficiency improved by 4–6 times. Endogenous U3 and U6 promoters driving sgRNA expression achieved up to 85% transformation rate of *MdPDS* gene for albino phenotypes [72, 74].

In addition, we observed that the CRISPR/Cas9 system driven by endogenous promoters in walnut induced a greater diversity of mutation types, including missense mutations (m) and nonsense mutations (n). These mutation types directly influenced the phenotype, especially the critical role of nonsense mutations (nn) in the albino phenotype. In contrast, when using exogenous promoters, mainly heterozygous mutations were observed, with fewer homozygous double-allele mutations. Charrier et al. observed similar results in apple, where endogenous promoters not only achieved higher editing efficiency but also generated a higher proportion of double-allele mutations [72]. A similar phenomenon was also observed in birch, where endogenous promoters led to higher editing efficiency and more diverse mutation types in the T_0_ generation [25]. These studies consistently demonstrate that the species-specific superiority of endogenous promoter-driven CRISPR systems in tree editing. Species-specific Pol III promoters can enhance sgRNA levels, significantly improve gene-editing efficiency, and show great potential in expanding mutation types and increasing the frequency of nonheterozygous mutations.

In summary, this study re-evaluated the walnut regeneration system and developed a more stable and efficient regeneration material. This advancement provides a solid technical foundation for genetic transformation and functional gene research in walnut (Fig. 5). Furthermore, we identified effective and robust endogenous Pol III promoters for walnut, which significantly enhanced the editing efficiency of the CRISPR/Cas9 system. These promoters lay the groundwork for establishing a multiplex genome-editing platform in walnut, thereby accelerating the application of CRISPR/Cas9 technology in functional genomics, trait improvement, and molecular breeding.

## Materials and methods

### Plant materials

The walnut germplasms selected for screening receptor materials were sourced from germplasm conservation orchards in six regions of China, including Aksu (Xinjiang), Luoyang (Henan), Baoding (Hebei), Dalian (Liaoning), Jiacha (Tibet), and from collections maintained by the Chinese Academy of Forestry (Beijing). Walnut fruits were harvested approximately 50 days after the peak period of female flowering. For each germplasm, 50 immature fruits were collected from the outer middle canopy on the sunny side of the trees. Immediately after harvest, fruits were placed into preservation containers and stored under refrigerated conditions to maintain freshness. Additionally, leaves collected from 25-year-old ‘Linzaoxiang’ walnut trees were used for promoter cloning experiments. The vector used for genetic transformation verification was *p35S: Ruby*, which is maintained in the Pei laboratory. The scaffold vector used for gene editing was the *pSCI* vector, obtained from the Zheng laboratory at Northeast Forestry University [25], *pEASY-Blunt Zero* was purchased from TransGen Biotech.

### Induction of SE in walnut

The immature walnut fruits were rinsed with running water for 30 min, followed by sequential surface sterilization using 75% ethanol for 1 min and 2% sodium hypochlorite solution for 20 min. After washing with sterile water 3–5 times, the sterilized walnut fruits were opened in a sterile laminar flow hood. The immature zygotic embryos were extracted and cultured on an induction medium (DKW 5.32 g/L, sucrose 30 g/L, myo-inositol 0.1 g/L, plant agar 3 g/L, 6-BA 2 mg/L, pH = 5.7). The cultures were incubated under dark conditions at 23 ± 2°C. After two weeks of dark incubation, the SEs were transferred to a maintenance medium (DKW 5.32 g/L, sucrose 30 g/L, myo-inositol 0.1 g/L, plant agar 3 g/L, pH = 5.7) for further culturing. The SE development of different walnut germplasm was observed. In this experiment, approximately 50 young fruits were selected from each germplasm. The somatic embryo induction rate was calculated as the ratio of explants that successfully induced somatic embryos to the total number of explants.

SE induction rate (%) = (Number of responsive explants/Total number of explants) × 100.

### SE regeneration in walnut

The SEs were separated from the immature embryos and transferred to the maintenance medium, with media changes performed every 7 days. Proliferation rate, deformity rate, and regeneration time were recorded. After the maintenance culture, the mature SEs were placed in a desiccator containing saturated NH_4_·NO_3_ solution at 23 ± 2°C for dehydration treatment over 5–7 days, then transferred to a germination medium (based on maintenance medium with 6-BA 1 and IBA 0.01 mg/L) and incubated under 25 ± 2°C with a 16-h photoperiod and light intensity of 40 μmol·m^−2^·s^−1^. For each genotype, 50 SEs were selected, with three repetitions, and proliferation rate, deformity rate, regeneration time, germination rate, and germination time were recorded.

SE proliferation rate (%) = (Number of proliferative SEs/Total number of SEs) × 100%.

SE deformity rate (%) = (Number of deformed SEs/Total number of SEs) × 100%.

SE germination rate (%) = (Number of germinated SEs/Number of SEs subjected to dehydration treatment) × 100%.

### SE preculturing and transformation

Preculturing of SEs: SEs with intact structures and consistent developmental stages were selected for preculturing, which was carried out for approximately 30 days. Infection: The *Agrobacterium tumefaciens* strain GV3103, containing the target plasmid, was cultured overnight to an OD value of around 0.6. After centrifugation, the supernatant was discarded, and the bacterial pellet was resuspended in infection medium (liquid maintenance medium supplemented with 100 μM acetosyringone). The precultured walnut SEs were immersed in the prepared suspension at room temperature for 15 min. After the infection, the embryos were transferred onto sterile dry filter paper and allowed to air dry under a sterile laminar flow hood until no liquid residue remained on the surface of the embryos. Co-cultivation**:** The infected SEs were placed on co-cultivation medium (liquid maintenance medium supplemented with 100 μM acetosyringone), with the morphological lower end of the embryos facing down. The co-cultivation was carried out under dark conditions at 23 ± 2°C for 2–3 days. Sterile culture: After co-cultivation, the SEs were transferred to sterile culture medium (liquid maintenance medium supplemented with 300 mg/L Temetine) to eliminate residual *Agrobacterium* contamination. During the first week, the culture medium was replaced daily, and in the following 2–3 days, the medium was replaced every 2–3 days. After approximately 4 weeks, new SEs (SE regeneration) began to grow, completing the sterile culture process. Selection culture: The SEs were cultured on selection medium containing the appropriate antibiotic(s) for selection. For selection, the *p35S:Ruby* vector carried a hygromycin resistance gene, with selection performed on medium containing 200 mg/L hygromycin. The *pSCI* vector conferred Basta resistance, and selection was conducted using 0.001% Basta. The medium was replaced every 5–7 days. After approximately 4 weeks, new SEs appeared, and the positive rate of regenerated SEs was recorded. During the genetic transformation process, we refer to the somatic embryos infected with *Agrobacterium* as the E_0_ generation. The secondary embryos that develop on the surface of E_0_ somatic embryos after infection are designated as the E_1_ generation. Likewise, secondary embryos that form on the surface of E_1_ somatic embryos are referred to as the E_2_ generation, and so on.

### T-DNA insertion site analysis

Genomic DNA was extracted from four walnut somatic embryo samples, including one wild-type control and three randomly selected RUBY transgenic lines. DNA purity and integrity were assessed using a NanoDrop 2000 spectrophotometer and 1% agarose gel electrophoresis. Only samples with an OD_260/280_ ratio between 1.8 and 2.0, clear intact bands, and total DNA quantity over 2 μg were selected for sequencing. Genomic DNA was sheared to 200~300 bp fragments using a Covaris M220 ultrasonicator, and sequencing libraries were constructed with the Illumina Nextera DNA Library Prep Kit according to the manufacturer’s protocol. Paired-end sequencing (2 × 150 bp) was performed on an Illumina HiSeq 2500 platform at a target depth of 10X, generating at least 6 Gb of raw data per sample, with Q30 scores exceeding 90%. Raw reads were subjected to quality control using fastp, with removal of adapter sequences and low-quality reads. High-quality clean reads were retained for downstream analysis.

To identify T-DNA insertion sites, Bowtie2 was employed to align the sequencing reads against the T-DNA sequence, filtering for reads containing both genomic and vector-derived sequences while removing vector-only reads. The remaining reads were mapped to the walnut reference genome using BLAST to determine the precise T-DNA integration sites. Based on the insertion site analysis, specific PCR primers were designed using Oligo7, with forward and reverse primers flanking both the vector and genome junctions (Fig. 3C and Supplementary Table S5), yielding an expected amplification product of approximately 500 bp. PCR verification was conducted on four transgenic lines to confirm the accuracy of the insertion site determination. All primers were synthesized by Sangon Biotech (Shanghai, China).

### Promoter cloning

The gene sequences of *Arabidopsis thaliana* snRNAs, AtU3b (*AT5G53902*), and AtU6-26 (*AT3G13855*), were used as references for the identification of walnut U3 and U6 snRNA genes. The confidence interval was set to 1e-20, and the sequences were aligned against the walnut genome [31]. DNA fragments upstream of the transcription start sites of the identified genes, which contain two key transcription elements (TATA-box and USE), were amplified. These fragments were then cloned into the *pEasy-Blunt Zero* vector for sequencing. Correctly sequenced plasmids were retained for use as templates in subsequent experiments.

### Vector construction

To identify effective PDS gene targets in walnut, sgRNAs were designed using CRISPR-p (http://cbi.hzau.edu.cn/crispr/) and CRISPR RGEN (http://www.rgenome.net/). These sgRNAs were optimized to minimize potential off-target cleavage sites and to achieve an appropriate GC content percentage, with a focus on matching the transcriptional features of the U3 and U6 promoters.

To evaluate the effectiveness of the JrU3 and JrU6 promoters in driving sgRNA expression, we linked the walnut PDS sgRNA to the JrU3 and JrU6 promoters via PCR. The JrU3/U6-PDSsgRNA expression cassette was then seamlessly cloned into the *pSCI* gene-editing vector. Additionally, we connected the *JrPDS* sgRNA to the AtU6-26 and BpU6-6 promoters to construct vectors with two exogenous promoters as controls.

### Identification and analysis of gene-edited lines

Gene-specific primers Cas9-F/R (Supplementary Table S5) were used to amplify the target region containing the edited sites, and transgenic plant genomic DNA was employed as the PCR template. The PCR products were cloned into an intermediate vector (*pEASY-Blunt Zero*), and Sanger sequencing was performed to obtain mutation information at the target sequence. The Sanger sequencing results were aligned with the reference sequence to analyze the editing efficiency and mutation types of sgRNAs driven by different promoters at the target loci.

### Data processing

Statistical analysis was performed using SPSS Statistics 25.0. Descriptive statistics, including means and standard deviations, were calculated. Differences among multiple groups were analyzed using one-way analysis of variance (ANOVA), followed by Tukey’s *post hoc* test to assess pairwise comparisons. Statistical significance was defined as *P* < 0.05. Graphs were generated using GraphPad Prism 9.5.0. DNA sequence alignment and analysis were carried out using DNAMAN 5.0 and SnapGene 4.2.4 software. DNAMAN 5.0 was used for multiple sequence alignment and consistency analysis, while SnapGene 4.2.4 was used for precise localization and sequence verification of target gene-editing sites and vector constructs, ensuring the accuracy and reproducibility of the results.

## Supplementary Material

Web_Material_uhaf187

## Data Availability

All the data generated in this study are included in the published article and its supplementary information.
