# Supplementary material for: Walnut genome editing: an optimized CRISPR/Cas9 platform with superior genotypes and endogenous promoters
Source: Hortic Res. 2025 Jun 24;12(10):uhaf187. doi: 10.1093/hr/uhaf187 (PMC12539868; doi:10.1093/hr/uhaf187)
Supplement: Web_Material_uhaf187 [file web_material_uhaf187.zip › Supplementary Figure.docx]

**Supplementary Figure** 1. Diagram of the entire somatic embryo induction process for Type II, III, and IV immature embryos.

Bar=0.5cm

**Supplementary Figur**e 2. Validation of the reporter gene 35S:Ruby in walnut genetic transformation

A. Transient expression of 35S:Ruby in walnut somatic embryos, showing light red pigmentation limited to the surface of immature embryos;

B. Transformation efficiency assessment: three independent experiments were conducted, each using 30 walnut somatic embryos as recipient material. Efficiency was calculated as the number of embryos exhibiting the RUBY phenotype divided by the total number of recipient embryos;

C. Stable genetic expression of 35S:Ruby in walnut somatic embryos. Upon magnification, the cotyledon surface reveals that secondary embryos also display the characteristic RUBY pigmentation.

**Supplementary Figure** 3. U6/U3 snRNA promoter sequence alignment analysis

A. Multiple sequence alignment of U3snRNA gene between walnut and *Arabidopsis thaliana*;

B. Multiple sequence alignment of U6snRNA gene between walnut and *Arabidopsis thaliana*;

Black lines indicate snRNA transcripts. The conserved elements USE(upstream sequence element) and TATA-like box are indicated by black lines. The “A" and “G”are shown, respectively, in the U3 and U6 promoters.

Supplementary Figure 4. Screening and identification of the phytoene desaturase (*PDS*) gene in walnut. In C, the gray boxes indicate exons; the black lines represent introns.

**Supplementary Figure** 5. Construction of endogenous U3 and U6 promoter gene editing vectors in walnut. Arrows represent promoters, and gray boxes represent sgRNAs

**Supplementary Figure** 6. Diagram of vector modification and sgRNA ligation site

Supplementary Figure 7. Editing status of *JrPDS* mediated by different promoters

Red represents the target sequence, gray represents Pam, blue lowercase represents base substitutions, white represents missing bases, and black represents inserted bases

Supplementary Figure 8. Statistics of all *JrPD*S editing typess

Supplementary Figure 9. All amino acid variant types after *JrPDS* gene editing.

The red font represents the base that has been replaced, and the blue represents the entire amino acid sequence that has been mutated
